# A Cadaveric Study to Define the Variant Patterns of Median Nerve Formation

**DOI:** 10.7759/cureus.39806

**Published:** 2023-05-31

**Authors:** Sumit Patil, Bertha Rathinam, Brijesh Kumar, Prashant Chaware, Naina Wakode, Santosh Wakode, Kusum Gandhi

**Affiliations:** 1 Anatomy, All India Institute of Medical Sciences, Bhopal, IND; 2 Anatomy, Atal Bihari Vajpayee Government Medical College, Vidisha, IND; 3 Physiology, All India Institute of Medical Sciences, Bhopal, Bhopal, IND

**Keywords:** axilla dissection, median nerve variations, musculocutaneous nerve, brachial plexus, nerve injury

## Abstract

The median nerve is one of the important nerves of the upper limb which supplies the muscles of the front of the forearm, muscles of the hand, and skin of the hand. Many works of literature mentioned its formation by the fusion of two roots, the medial root from the medial cord and the lateral root from the lateral cord. But multiple variations in the median nerve formation have clinical importance from surgical and anesthetic points of view. For the study purpose, we dissected 68 axillae of 34 formalin-fixed cadavers. Out of 68 axillae, two (2.9%) showed median nerve formation by a single root, 19 (27.9%) showed median nerve formation by three roots, and three (4.4%) showed median nerve formation by four roots. A normal pattern of median nerve formation by fusion of two roots was seen in 44 (64.7%) axilla. The knowledge of variant patterns of median nerve formation will be helpful to surgeons and anesthetists while performing surgical or anesthetic procedures in the axilla to avoid any injury to the median nerve.

## Introduction

The median nerve (MN) is formed by the fusion of the medial root (C8, T1) and lateral root (C6, C7) in front of the third part of the axillary artery. The MN supplies the majority of muscles of the front of the forearm and hand, it also innervates the palmar skin of lateral three-and-a-half fingers and the skin at the nail bed at the dorsum of the lateral three-and-half fingers. Fibers from the lateral root of the median nerve (LRM) innervate the palmar skin of the thumb, index, and lateral half of the middle finger and pronator teres, flexor carpi radialis, and some part of flexor digitorum superficialis. The medial root of the median nerve (MRM) innervates the skin of the medial side of the middle finger and lateral side of the ring finger and also supplies to palmaris longus, flexor digitorum superficialis, lateral part of flexor digitorum profundus, flexor pollicis longus, pronator quadratus and median innervated muscles in hand [1]. As each root of the median nerve has a specific area of innervations, the variant patterns of roots gained clinical importance.

The normal pattern of MN formation from two roots has been reported to vary from 48- 88.3% [2,3]. Whereas its formation from three or four roots has been reported in 3-40% of cases [4,5]. The most common variations in the roots of MN were seen in the lateral root and it was reported to be thinner than the medial root of MN. The nerve fibers which had not contributed lateral root entered the musculocutaneous nerve (MCN) and get reentry in MN through the communicating branches [6]. Morphological variations of median nerve formation and its communication with other nerve bundles in the axilla and root of the neck have clinical importance from the anesthetic and surgical points of view.

## Materials and methods

The study was conducted in the Department of Anatomy, All India Institute of Medical Sciences (AIIMS) Bhopal in the central region of India. For the study, formalin-embalmed 34 cadavers (22 male and 12 female) of age 40-92 years were utilized. The cadavers with deformity or trauma in the axilla or arm were excluded from the study. The dissection steps of Cunningham’s Manual of Practical Anatomy [7] were followed to dissect 68 upper extremities (34 left and 34 right). The skin, superficial fascia, and deep fascia of the axilla and arm were reflected. The muscles were retracted to get a visualization of the brachial artery and median nerve (MN). The MN being the most superficial prominent nerve of the arm was easily identified. It was traced upwards in the axilla to find its roots which usually came from the medial and lateral sides of the axillary artery. The MN formation in relation to the axillary artery by its medial root (MRM) and lateral root (LRM) was noticed. An attempt was done to find the additional bundles of nerve fibers joining to MN, MRM, or LRM. The observations were noted in the pro-forma sheet according to cadaver number, sex, and side of the axilla. The prominent variant patterns of MN formation were digitally photographed.

## Results

The usual pattern of the MN formation by the fusion of two roots, MRM and LRM was seen in 44 (64.7%) of upper limbs. In this study, we also observed variant patterns of MN formation from one, three, and four roots as shown in Table 1. The variant MN formation and normal pattern were compared in male and female cadavers by applying Fisher Exact Test. The statistic value of the test was 0.2883 which indicated a non-significant result (p>0.05). 

**Table 1:** Different patterns of median nerve formation.

	1 Root		2 Roots		3 Roots		4 Roots	
	Male	Female	Male	Female	Male	Female	Male	Female
Left	2	0	11	9	7	3	2	0
Right	0	0	15	9	6	3	1	0
Total	2	0	26	18	13	6	3	0
Gross Total	2 (2.9%)		44 (64.7%)		19 (27.9%)		3 (4.4%)	

One root

**Figure 1:**
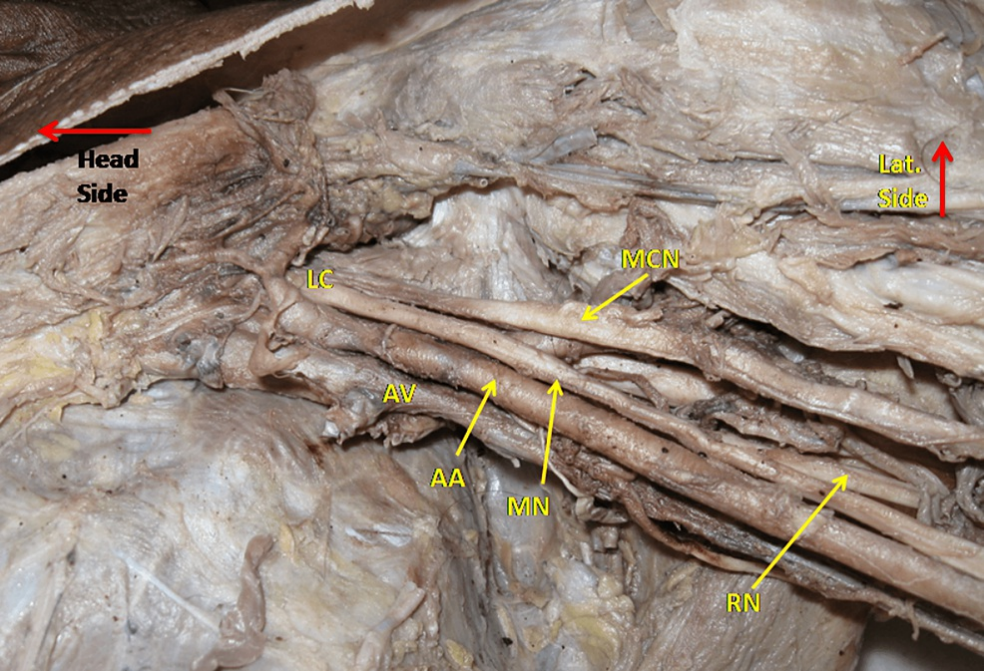
Shows left axilla with Median nerve (MN) formation from a single root. Here lateral cord (LC) continues as MN after giving musculocutaneous nerve (MCN). AA- Axillary artery, AV- Axillary vein, RN- Radial nerve

The MN formation from a single root was observed in two (2.9%) cases out of 68 upper limbs. Both cases were from male cadavers and on the left side. The lateral cord after giving the lateral pectoral nerve is divided into two nerves, MN and musculocutaneous nerve (MCN). MN did not receive a contribution from the medial cord through MRM. In relation to the second and third parts of the axillary artery, both the MN and MCN were on the lateral side as shown in Figure 1. 

Three roots

**Figure 2:**
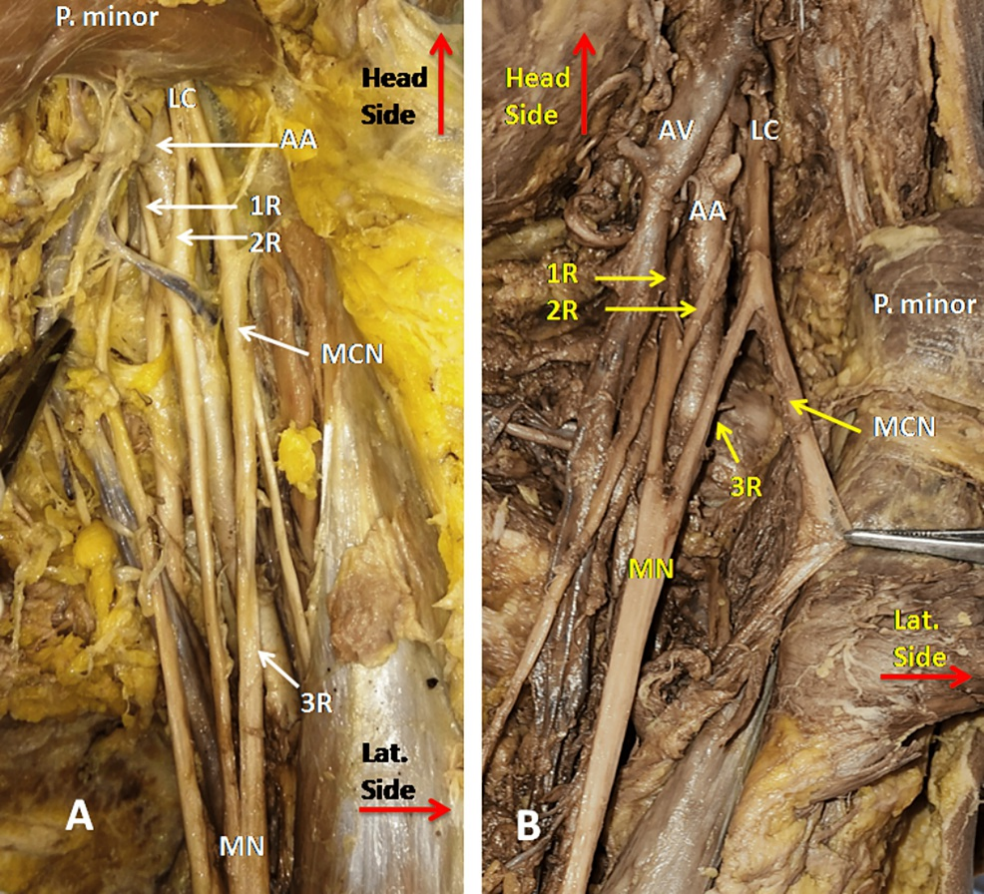
Both figures A & B of left axilla of different cadavers show the formation of Median nerve (MN) from three roots. In figure A, third root (3R) arise from the musculocutaneous nerve (MCN) and in figure B, 3R arise from the lateral cord (LC) of brachial plexus. Pectoralis minor muscle (P. minor) was used as reference for location of LC. AA- Axillary artery, AV- Axillary vein, 1R- 1st root -medial root of median, 2R- 2nd root.

**Figure 3:**
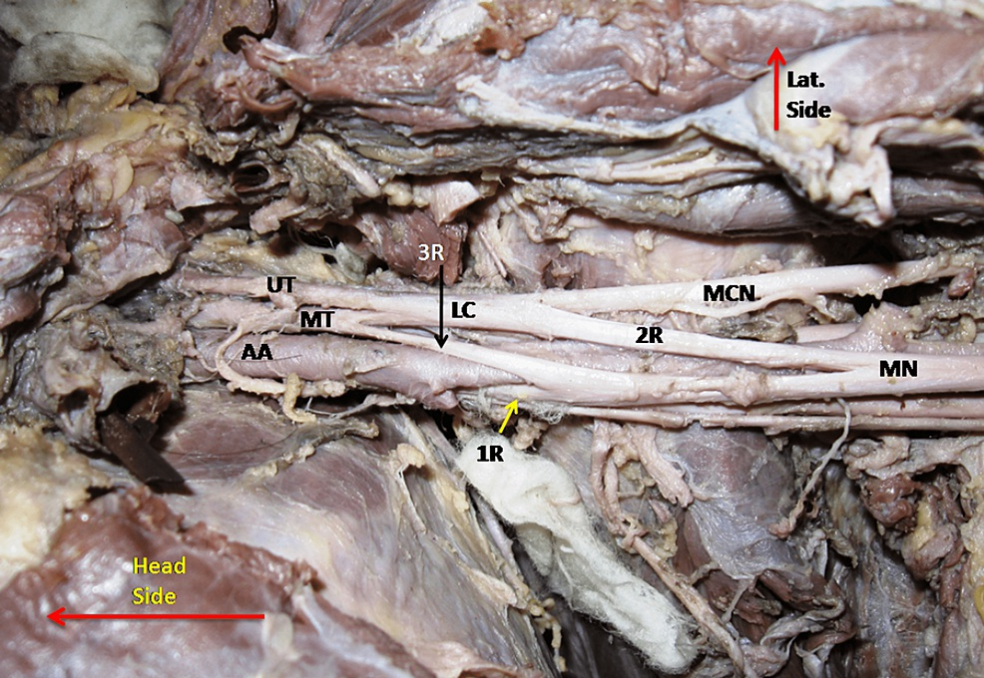
Showing the formation of median nerve (MN) from the three roots, and 3rd root (3R) arise from the middle trunk (MT) of brachial plexus. AA- Axillary artery, LC- Lateral cord, MCN- Musculocutaneous nerve, UT- Upper trunk, 1R- 1st root, 2R- 2nd root.

The MN formation by three roots was found in 19 (27.9%) upper limbs, 13 male and six female. The additional third root was coming from the lateral side either upside or down to the LRM. The 3rd root arose from MCN in 10(14.7%) cases (Figure 2A) and from the lateral cord in eight (11.7%) cases (Figure 2B). In one case (1.5%), the third root was seen coming from the middle trunk as shown in Figure 3. 

Four roots

**Figure 4:**
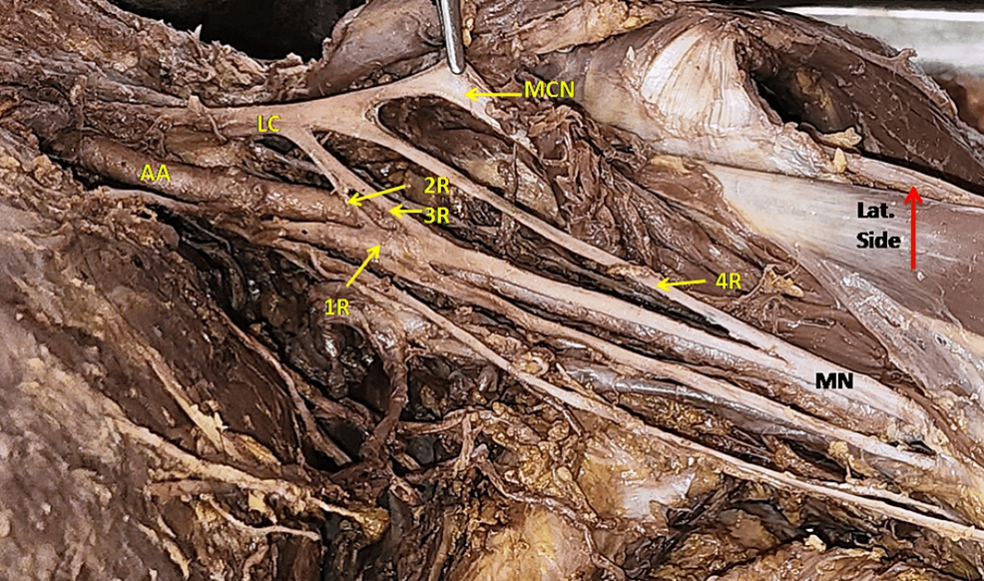
Showing the formation of Median nerve (MN) from 4 roots. 1st root (1R) arise from the medial cord while the 2nd , 3rd and 4th root (2R,3R,4R) arise from the lateral cord (LC). AA- Axillary artery, MCN- Musculocutaneous nerve.

**Figure 5:**
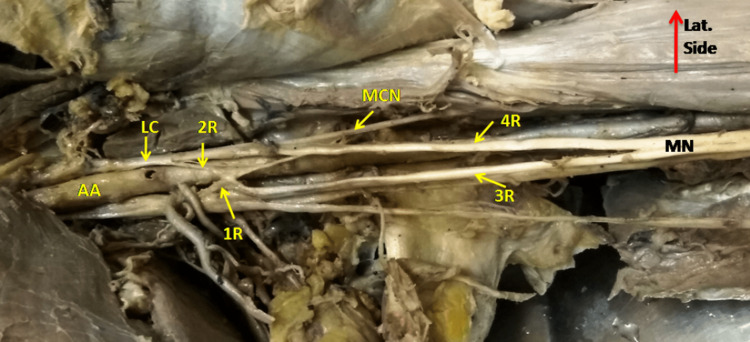
Showing the formation of Median nerve (MN) by 4 roots. Here 1st root (1R) arises from the medial cord and join the 4th root (4R) arising from the lateral cord (LC) and 2nd root (2R) arises from the LC and join the 3rd root (3R) arising from medial cord. AA- Axillary artery, MCN- Musculocutaneous nerve.

The MN formation by four roots was observed in three (4.4%) upper limbs of male cadavers. Two types of patterns were observed in the formation of the median nerve by roots. At first, the two additional roots were coming from the lateral side of the axillary artery along with LRM from the lateral cord and MCN (Figure 4). In the second pattern, 2-2 roots came from the medial cord and lateral cord, out of which smaller roots from each side crossed the axillary artery and merged on the opposite side larger root forming an ‘X’ anterior to the axillary artery as shown in Figure 5. The first pattern was seen in two cases and the second one in one case.

## Discussion

For the development of the upper limbs, somites from the lower cervical and upper thoracic regions elongate and carry their own spinal nerves to form the brachial plexus. The seventh cervical segmental artery which gives rise to the axillary artery passes in between the medial and lateral cords of the brachial plexus. If the subclavian-axillary artery stem develops from the sixth or eighth segmental artery, the variations in the relationship between the axillary artery and brachial plexus cords can be seen [8]. We observed that the MN was formed from a single root, two roots, three roots, and four roots. In the variant patterns of MN formation, the main contribution was from the MCN as an additional root or merging of MN and MCN. So for the interpretation of the relation between MCN and MN, the phylogenetic and comparative anatomy view can be considered. Kosugi et al. [9] discussed that there was only one trunk in the upper limb of the lower vertebrates which was equivalent to the MN and the communication between MN and MCN was also seen in mammals like dogs. 

Woźniak et al. [10] reported 5 (2.3%) cases out of 220 in which the median nerve was formed by a single root as a continuation of the lateral cord. We found 2 (2.9%) of MN formed from the single root which was exclusively from the lateral cord. 

Akhtar et al. [11] reported median nerve formation by three roots in 25% of male limbs among which the third root was contributed from the lateral cord in 16.07% of cases and from MCN in 8.93% of cases. While in females three roots were found in 21.42% of cases and the third root arose from the lateral cord in 14.28% of cases and from the MCN in 7.14% of cases. We found MN formation by three roots in 19 (27.9%) upper limbs out of which 13 were male and six the female cadavers. The additional third root was coming from MCN in 10(14.7%) cases and from the lateral cord in eight (11.7%) cases. In one case (1.5%), it was coming from the middle trunk. Other studies reported median nerve formation by three roots in 11.6 to 22.4 % of cases [12-16].

We observed MN formation by four roots in 4.4% of cases. The same finding was also reported by Ghosh et al. [15] in 5% of cases, Budhiraja et al. [16] in 3.57% of cases, and Hada et al. [17] in 2% of cases. Natsis et al. [18] reported a case of MN formed by five roots, in which the additional three roots originated from the lateral cord. We did not find any MN formed from five roots. 

This study was limited to the MN formation in the axilla and arm region only. The distribution of the MN and its relations with the other neurovascular structures were excluded from the study. 

## Conclusions

In the majority of cases, MN was formed by two roots, one from the lateral cord and another from the medial cord of the brachial plexus. However, in a few cases, MN was observed to be formed by a single root as a continuation of the lateral cord. The MN may be formed by one to four roots, out of this, the formation of MN by three roots was the commonest variant pattern. This information will be helpful in the diagnosis of MN injury. Such different patterns of MN formation should be kept in mind while performing regional anesthetic blocks. This information will also help the surgeons during surgery of the axilla region to avoid any injury to the additional root of MN. The additional roots of MN may increase the chances of compression of the axillary artery.

## References

[1] Standring S (2016). Gray’s anatomy: the anatomical basis of clinical practice.

[2] Fazan VPS, Amadeu AdS, Caleffi AL, Filho OAR (2003). Brachial plexus variations in its formation and main branches. Acta Cir Bras.

[3] Nasr AY (2012). Morphology and clinical significance of the distribution of the median nerve within the arm of human cadavers. Neurosciences Riyadh.

[4] Pandey SK, Shukla VK (2007). Anatomical variations of the cords of brachial plexus and the median nerve. Clin Anat.

[5] Sargon MF, Uslu SS, Celik HH, Akşit D (1995). A variation of the median nerve at the level of brachial plexus. Bull Assoc Anat Nancy.

[6] Hollinshead WH (1982). General survey of the upper limb. Anatomy for Surgeons, The Back and Limbs.

[7] Romanes GJ (1986). Upper and lower limbs. Cunningham’s manual of practical anatomy.

[8] Hollinshead WH (1982). Pectoral region, axilla and shoulder. Anatomy for Surgeons. The Back and Limbs.

[9] Kosugi K, Shibata S, Yamashita H (1992). Supernumerary head of biceps brachii and branching pattern of the musculocutaneus nerve in Japanese. Surg Radiol Anat.

[10] Woźniak J, Kędzia A, Dudek K (2012). Anatomical variability of median nerve formation in human foetuses in clinical aspect. Adv Clin Exp Med.

[11] Akhtar MJ, Kumar S, Chandan CB, Kumar B, Sinha RR, Akhtar MK, Kumar A (2022). Variations in the formation of the median nerve and its clinical correlation. Maedica (Bucur).

[12] Pattanshetti SV, Jevoor PS, Shirol VS, Dixit D, Bhimalli S (2012). A study of the formation and branching pattern of brachial plexus and its variations in adult human cadavers of north Karnataka. Journal of the Scientific Society.

[13] Priya A, Gupta C, D'souza A S (2019). Cadaveric study of anatomical variations in the musculocutaneous nerve and in the median nerve. J Morphol Sci.

[14] Passey J, Rabbani P, Razdan SK, Kumar S, Kumar A (2022). Variations of median nerve formation in North Indian population. Cureus.

[15] Ghosh B, Dilkash MN, Prasad S, Sinha SK (2022). Anatomical variation of median nerve: cadaveric study in brachial plexus. Anat Cell Biol.

[16] Budhiraja V, Rastogi R, Asthana AK (2012). Variations in the formation of the median nerve and its clinical correlation. Folia Morphol (Warsz).

[17] Hada S, Kadel M, Pandit TK, Basnet KS (2020). Variations in formation of median nerve: a cadaveric study. Journal of Chitwan Medical College.

[18] Natsis K, Paraskevas G, Tzika M (2016). Five roots pattern of median nerve formation. Acta Medica (Hradec Kralove).

